# The RCAN1.4-calcineurin/NFAT signaling pathway is essential for hypoxic adaption of intervertebral discs

**DOI:** 10.1038/s12276-020-0441-x

**Published:** 2020-05-29

**Authors:** Bao Huang, Yongqing He, Shengwen Li, Xiaoan Wei, Junhui Liu, Zhi Shan, Yue Huang, Jian Chen, Fengdong Zhao

**Affiliations:** 1 Department of Orthopedic Surgery, Sir Run Run Shaw Hospital, Zhejiang University School of Medicine, Hangzhou, China; 2 Key Laboratory of Musculoskeletal System Degeneration and Regeneration Translational Research of Zhejiang Province, Hangzhou, China; 3 Department of Orthopedic Surgery, Haining People’s Hospital, Haining, China

**Keywords:** Transcriptional regulatory elements, Extracellular signalling molecules

## Abstract

Calcipressin-1, also known as regulator of calcineurin 1 (RCAN1), can specifically bind calcineurin at or near the calcineurin A catalytic domain and downregulate calcineurin activity. However, whether RCAN1 affects the hypoxic intervertebral disc (IVD) phenotype through the calcineurin/NFAT signaling pathway remains unclear. First, we confirmed the characteristics of the degenerative nucleus pulposus (NP) by H&E, safranin O/fast green and Alcian blue staining, and detected increased RCAN1 levels in the degenerative NP by immunohistochemistry. Then, we demonstrated that the protein level of RCAN1.4 was higher than that of RCAN1.1 and progressively elevated from the control group to the Pfirrmann grade V group. In vitro, both hypoxia (1% O_2_) and overexpression of HIF-1α reduced the protein level of RCAN1.4 in rat NP cells in a dose- and time-dependent manner. We further found that miRNA-124, through a nondegradative pathway (without the proteasome or lysosome), suppressed the expression of RCAN1.4. As expected, calcineurin in NP cells was activated and primarily promoted nuclear translocation of NFATc1 under hypoxia or RCAN1.4 siRNA transfection. Furthermore, SOX9, type II collagen and MMP13 were elevated under hypoxia, RCAN1.4 siRNA transfection or NFATc1 overexpression. Using chromatin immunoprecipitation (ChIP) and a luciferase reporter assay (with mutation), we clarified that NFATc1 increasingly bound the SOX9 promotor region (bp −367~−357). Interaction of HIF-1α and NFATc1 promoted MMP13 transcription. Finally, we found that FK506 reversed hypoxia-induced activation of the calcineurin/NFAT signaling pathway in NP cells and an ex vivo model. Together, these findings show that the RCAN1.4-calcineurin/NFAT signaling pathway has a vital role in the hypoxic phenotype of NP cells. RCAN1.4 might be a therapeutic target for degenerative disc diseases.

## Back pain: overactive protein causes disc breakdown

Treatments targeting a protein that is overexpressed in damaged spinal cartilage could ease degenerative conditions associated with lower back pain. The intervertebral discs are complex cartilage tissues that absorb forces while allowing the motion of our spines. An immune-promoting enzyme called calcineurin is important in maintaining the supple, gel-like structure of the central part of each disc, the nucleus pulposus (NP). Fendong Zhao and Jian Chen at Zhejiang University School of Medicine Hangzhou, China and co-workers showed that RCAN1.4, a protein known to suppress calcineurin, is overexpressed in damaged human NPs. The team further revealed how a signaling pathway starting with RCAN1.4 suppresses key genes involved in forming the collagen fibers that hold the NP together. They therefore suggest that therapies targeting this protein could benefit patients suffering from disc degeneration diseases.

## Introduction

Intervertebral disc degeneration (IVDD) is a leading cause of low back pain (LBP), one of the most common clinical complaints^1,2^. Intervertebral discs (IVDs) include the central hydrated chondrocyte-like nucleus pulposus (NP), which is inferiorly and superiorly bounded by the cartilaginous endplate (CEP) and laterally surrounded by the concentric lamellae of the annulus fibrosus (AF). IVDs are a complex tissue that accommodates high biomechanical forces and permits a range of motions between adjacent vertebrae^3^. Notably, the high proteoglycan content in the central water-binding NP is an important component of IVDs, which absorb stress and maintain the structure and function of the spine^4^. In addition, because of injury and inflammatory factors infiltration, imbalance between extracellular matrix anabolism and catabolism in the NP can lead to degenerative disc diseases^5^. Human IVDs are the largest nonvascular tissue in the human body^6,7^. Therefore, the central NP is completely hypoxic and expresses hypoxia inducible factor 1 alpha and 2 alpha (HIF-1α and HIF-2α)^8^. HIF-1α promotes ECM synthesis in NP and maintains the intracellular pH and viability of NP cells^9^. However, the protein level of HIF-1α in the degenerative disc is generally reduced^10–12^.

Calcipressin-1, also known as regulator of calcineurin 1 (RCAN1)^13^, is in a family of negative endogenous regulators of calcineurin activation and can specifically bind the calcineurin A catalytic domain and downregulate calcineurin activity^14,15^. Calcineurin, composed of calcineurin A (the catalytic domain, CnA) and calcineurin B (the binding domain, CnB), is a protein phosphatase enzyme that primarily promotes the nuclear translocation of NFAT. The RCAN1 gene includes seven exons, and alternative splicing generates two main isoforms: RCAN1.1 (252 amino acids, 39 kDa) and RCAN1.4 (192 amino acids, 29 kDa). Recent studies showed that RCAN1.4 has a critical role in cancer growth^16,17^, endothelial cells migration^18,19^ and neuronal apoptosis^20^. Another study showed that prolyl hydroxylase domain 2 (PHD2) deficiency, which contributed to the limited oxygen-dependent degradation of HIF-1α and might serve as a chronic HIF-1α stabilization model, promotes skeletal muscle fiber repair through a calcineurin/NFATc1-dependent pathway^21,22^. However, whether RCAN1.4 affects the hypoxic IVD phenotype through the calcineurin/NFAT signaling pathway remains unclear.

Previous studies showed that the calcineurin/NFATc1 signaling pathway regulates sex-determining region Y (SRY)-box 9 (SOX9) expression during tracheal chondrogenesis^23^ and that NFATc1 can induce SOX9 transcription in the pancreas and chondrocytes^23–25^. SOX9 is vital for aggrecan and typeIIcollagen gene expression. Furthermore, HIF-1α is a positive regulator of SOX9 activity^22,26^ and metabolically controls collagen synthesis and modification in chondrocytes^27^. In addition, HIF-1α is upregulated by a process that involves calcineurin/NFAT in activated mast cells^28^, and HIF-1α participates in the activation of calcineurin/NFAT^29^. However, the roles of RCAN1 in ECM synthesis and remodeling of the IVD have not been elucidated. Therefore, we hypothesize that the interaction between HIF-1α and the RCAN1.4-calcineurin/NFAT signaling pathway promotes ECM synthesis and remodeling to preserve the IVD phenotype.

To test our hypothesis, we detected the gene and protein expression levels of RCAN1.1 and RCAN1.4 in human NP specimens and analyzed their correlation with disc degeneration. Further, we evaluated the role of the RCAN1.4-calcineurin/NFAT signaling pathway in the hypoxic NP cell phenotype and silenced RCAN1.4 to detect its role in the NP cells. Moreover, we determined that FK506 (a calcineurin inhibitor) repressed hypoxia-induced activation of the calcineurin/NFAT signaling pathway using in vitro and ex vivo models.

## Materials and methods

### Human tissue from normal and degenerated NP

Informed consent was obtained from each patient. Our study was approved by the Ethical Review Board of Sir Run Run Shaw Hospital. From June 2018 to July 2019, human lumbar NP tissues were obtained from patients with vertebral fractures who did not present degenerative changes based on MR images, or patients with LBP who were undergoing discectomy and fusion. The patient information is listed in Supplemental Table 1. According to the Pfirrmann grading system^30^, the lumbar disc specimens were further subdivided into Pfirrmann grade I/II, grade III, grade IV, and grade V groups. Specimens were carefully dissected and either fixed in 4% paraformaldehyde for 48 h at 4 °C or immediately frozen in liquid nitrogen.

### NP cell isolation and treatment

The animal experiments were approved by the Ethical Review Board of Sir Run Run Shaw Hospital (affiliated with Zhejiang University, Hangzhou, Zhejiang). All Sprague–Dawley rats (male, 150–200 g, 8-week-old) used were provided by the Animal Center of Zhejiang University (Zhejiang, China). Human or rat NP tissues were isolated from lumbar discs and then cut into small pieces (<1 mm^3^ in size) as quickly as possible as previously described^31^. After incubation with type II collagenase (0.2 mg/mL) (Sigma-Aldrich, USA) for 2 h at 37 °C, the cells were harvested and centrifuged at 800 rpm for 5 minutes. The cells were resuspended with Dulbecco’s modified Eagle’s medium with 20% fetal bovine serum (Gibco, USA), 1 mm sodium pyruvate, 2 mm glutamine, and 1% penicillin/streptomycin and cultured in a humidified atmosphere containing 5% CO_2_ at 37 °C. RCAN1 or HIF-1α expression was evaluated by treating NP cells with hypoxia, dimethyloxalylglycine (DMOG, 0.5 μm and 1 μm) or RCAN1.4 siRNA (Vigenebio, Shandong, China). The hypoxic incubator contained 1% O_2_, 5% CO_2_, and 94% N_2_. In brief, using Lipofectamine 3000, NP cells at an up to approximately 70% confluence were transfected with negative control siRNA or target siRNA (100 nm) (Invitrogen, CA, USA). To mimic hypoxia, we stimulated NP cells with DMOG (a competitive PHD inhibitor) (Sigma-Aldrich) for the indicated duration. Following the treatment, we used TRIzol reagent (Invitrogen) or RIPA (Gibco, Gaithersburg, MD, USA) to extract total RNA or proteins.

### RNA isolation and quantitative RT-PCR

Human NP tissues were finely powdered with a sterilized mortar and pestle. Total RNA was extracted using the Ultrapure RNA Kit (CW0581, CWBIO, China). A NanoDrop 2000 was used to measure RNA quantity. Complementary DNA was synthesized using PrimeScript RT MasterMix (Takara Bio, Otsu, Japan). SYBR Green qPCR Master Mix (Takara Bio, Otsu, Japan) was used to quantify transcript levels. Sequences of the RT-qPCR primers are summarized in Supplemental Table 2. Experimental reactions were conducted by preincubation (95 °C for 5 min), amplification (95 °C for 15 s, 60 °C for 60 s for 40 cycles), melting curve analysis (95 °C for 15 s, 60 °C for 60 s), and cooling (40 °C for 5 minutes). RT-qPCR was performed in triplicate, and amplification signals were normalized to the β-actin signal in the same reaction.

### Western blotting

Following treatment, ground NP tissues or NP cells were immediately placed on ice and incubated for ~40 min in RIPA buffer (Gibco) with 100 mm phenylmethanesulfonyl fluoride (PMSF) (Beyotime, Zhengzhou, China) and protease inhibitor cocktail (PIC) (Millipore, USA), followed by centrifugation at 12,000 × *g* for 15 min. Proteins were resolved by 10% sodium dodecyl sulfate-polyacrylamide gel electrophoresis (SDS-PAGE) and transferred to polyvinylidene difluoride membranes (Millipore, Billerica, MA) by electroblotting. The membranes were incubated in 5% (w/v) nonfat dry milk in TBST (1‰ Tween20) at RT for 1–2 h and then incubated with anti-RCAN1 (ab140131, Abcam, Cambridge, MA; abs121164, absin, China), anti-HIF-1α (36169, CST; R1510-5, Huabio, Hangzhou), anti-SOX9 (ab185966, Abcam; ET1611-56, Huabio), anti-MMP13 (ab39012, Abcam), anti-type II collagen (ab34712, Abcam), anti-NFATc1 (8032, CST; ET1704-45, Huabio), anti-histone H3 (4499, CST), anti-ubiquitin (3936, CST), anti-tubulin-α (Proteintech, USA), or anti-β-actin (Beyotime) and immunoglobulin G antibodies (1:1000 dilution) at 4 °C overnight. After 5 × 7 min of TBST washing, protein bands were incubated with horseradish peroxidase (HRP)-conjugated goat anti-rabbit/mouse immunoglobulin G (1:5000 dilution; CST), followed by detection using electrochemical luminescence reagent (Millipore, Billerica, MA). Bands were detected with Image Lab software (Bio-Rad, Hercules, CA). The obtained images were measured with the ImageJ software (National Institutes of Health, Bethesda, MD, USA)

### Immunohistochemistry (IHC)

Specimens were embedded in paraffin and sliced into 4 μm-thick sections. IHC was conducted with an SP Rabbit & Mouse HRP Kit (CW2069, CWBIO). To investigate the immunoreactivity of RCAN1 (ab140131, Abcam) or HIF-1α, slices were immunostained at 4 °C overnight. Slices were incubated with rabbit anti-RCAN1 or anti-HIF-1α polyclonal antibodies and immunoglobulin G diluted 1:100 or 1:200. Three pathologists blinded to the clinical tissue data were responsible for counting the total cells and RCAN1- or HIF-1α-positive cells in three sections of each specimen at a high-power field (magnification ×200). The sections were recounted if the intraclass correlation coefficient was below 0.8.

### Immunofluorescence staining

Cells were placed on slides in six-well plates at a density of 1 × 10^4^ cells. After incubation overnight, the cells were stimulated with or without hypoxia or FK506 for the indicated duration. The slides were then fixed in 4% paraformaldehyde for 30 minutes and permeabilizated with 0.5% Triton-X100 for 30 minutes at RT. After incubation with 5% bovine serum albumin for 1 h, the slides were incubated with anti-SOX9, anti-type II collagen and anti-NFATc1 (1:100~200 dilution) at 4 °C overnight. Nuclei were stained with 0.1 μg/mL DAPI (Sigma-Aldrich) for 30 minutes at RT. Cells were imaged using fluorescence microscope model BX51TRF (Olympus Corporation, Tokyo, Japan). Images were analyzed by ImageJ software (National Institutes of Health).

### Immunoprecipitation (IP) assay

In brief, cellular lysates were immunoprecipitated into 2 μg of anti-RCAN1 or anti-Flag antibody (0912-1, Huabio) at 4 °C overnight, followed by incubation with 30 μL of protein A/G-agarose (50% v/v) for 3 h at 4 °C. After centrifugation at 13,000 × *g* for 12 min, protein complexes were harvested. After five washes with cold PBS, bound proteins were resolved by 10% SDS-PAGE and finally incubated with anti-Myc (R1208-1, Huabio) and anti-ubiquitin (3936, CST).

### Chromatin Immunoprecipitation (ChIP)

In brief, using a ChIP kit (9002, CST), NP cells were stimulated with or without hypoxia for 8 h, followed by incubation in 37% formaldehyde for 10 minutes, as described previously^32^. After cross-linking was terminated with glycine and the cells were washed 3 × 10 minutes using cold PBS with 1 mm PMSF and PIC, cells were harvested, resuspended in ChIP buffer and incubated for 30 minutes. The cells were then sonicated via 10 cycles consisting of 30-s pulses and 30-s intervals on an ice-water mixture. After centrifugation at 9400 × *g* for 10 minutes, the supernatants were diluted with ChIP dilution buffer with PIC. One percentage of the total chromatin DNA was used as input. After incubation with anti-RCAN1 or anti-Flag antibody at 4 °C overnight, the supernatants were collected and then precleared with protein A/G-agarose beads. By vortexing at 1200 rpm/min for 30 minutes at 65 °C, chromatin was eluted from agarose beads. To reverse crosslinks, chromatin was incubated with 6 µl of 5 m NaCl and 2 µl of proteinase K at 65 °C for 2 h. DNA for PCR analysis was purified using a DNA spin column.

### Luciferase assay

NP cells were seeded in a 12-well plate at a density of 1 × 10^4^ cells and transfected with or without equal amounts of pGL4 vector (Promega, WI, USA), SOX9-Luc2 plasmid, or MMP13-Luc2 plasmid (Sunya, Hangzhou, China) with NFATc1 or HIF-1α plasmid. Meanwhile, Renilla plasmid was cotransfected to normalize the transfection efficiency. After 24 h, a luciferase assay was then carried out using a dual-luciferase reporter assay system (Beyotime).

### **Isolation and ex vivo culture of rats lumbar IVDs**

Whole lumbar IVDs (NP, CEP, AF, and adjacent vertebral endplates) were gently harvested from rats (male, 150~200 g, 8 weeks old) as previously described^31^. They were cultured with DMEM, 50 μg/mL l-ascorbate and 10% fetal bovine serum and randomly divided into four groups and treated as follows (*n* = 6): (A) IVDs+DMSO, (B) IVDs+FK506 (1 μm), (C) IVDs+DMOG (1 μm), and (D) IVDs+DMOG (1 μm)+FK506 (1 μm). Treatments were administered for 2 weeks in six-well plates in a humid incubator (37 °C, 5% CO_2_). The expression levels of Sox9, type II collagen, MMP3, MMP13, ADAMTS4, and NFATc1 in the NP zone were measured using RT-qPCR and immunofluorescence staining.

### Histological analysis

Rat IVDs were fixed in 4% paraformaldehyde for 24 h, decalcified in 12% EDTA for 2 weeks, and then embedded in paraffin. For every IVD, slices (4 μm-thick) were obtained using a microtome (Leica, Germany). Slices were stained with safranin O/fast green or Alcian blue to examine cell morphology and ECM degeneration. Degenerative discs underwent detailed histological scoring (Supplemental Table 3) as previously described^31^. To examine RCAN1, MMP13 or type II collagen immunoreactivity, IHC or immunofluorescence staining was performed. Slices were photographed and assessed using a digital microscope.

### Statistical analysis

The data are presented as the mean and standard deviation. Data analyses were conducted using SPSS 19.0 (SPSS, Chicago, IL, USA). Statistical differences were analyzed by one-way analysis of variance or Student’s *t* test, followed by Tukey’s post hoc analysis when appropriate. Differences with a *P* value < 0.05 were considered statistically significant.

## Results

### High RCAN1.4 in expression degenerated human NP tissues

**Fig. 1:**
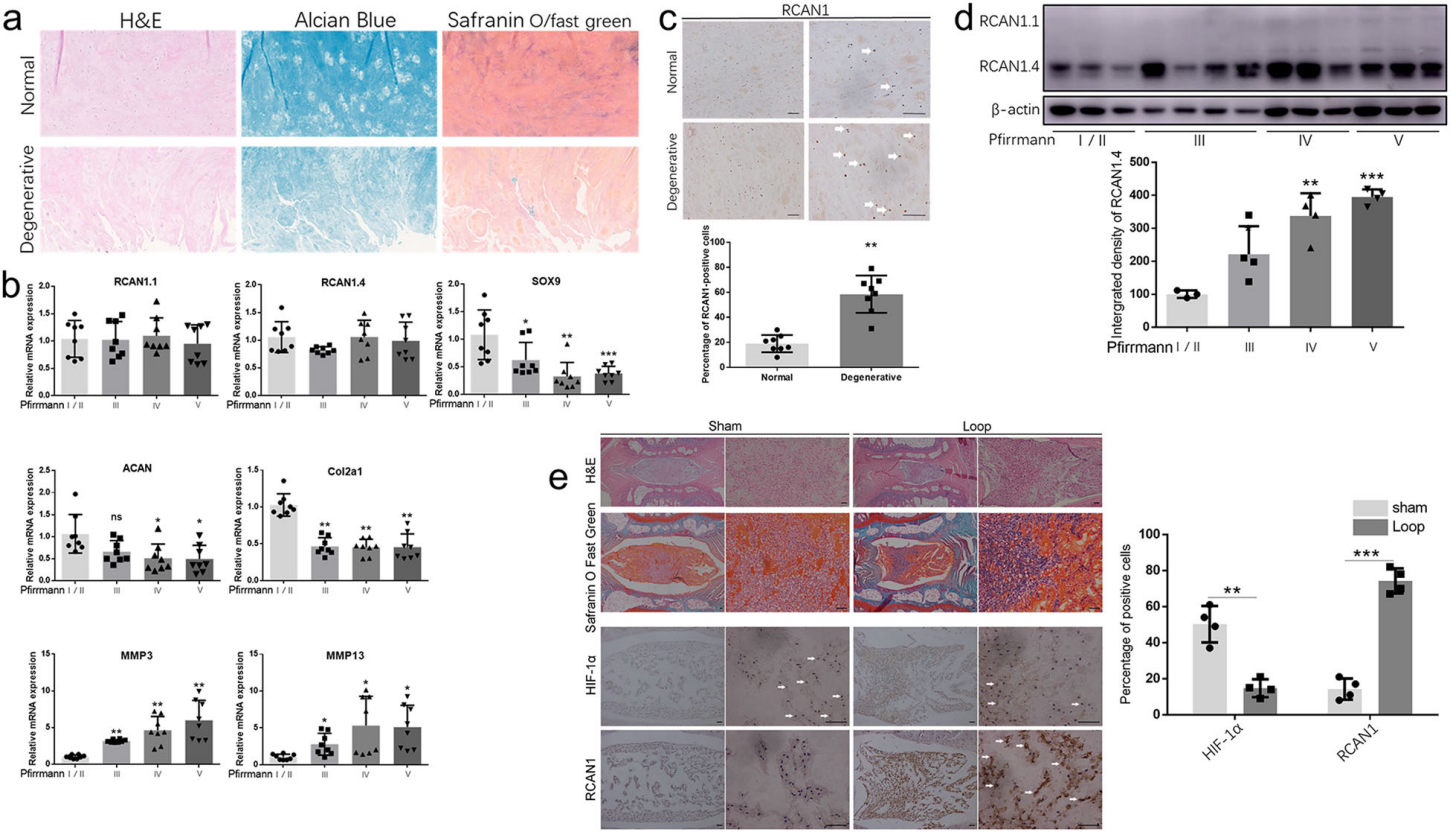
RCAN1.4 was upregulated in human degenerative NP samples. **a** Hematoxylin & eosin, safranin O/fast green, and Alcian blue staining of normal and degenerative human lumbar NP tissues is shown. **b** Gene expression of RCAN1.1, RCAN1.4 SOX9, type II collagen (Col2a1), aggrecan (ACAN), MMP13, and MMP3 in the Pfirrmann grade I/II, III, IV, and V groups (*n* = 8). **c** Cells immunopositive for RCAN1 in the human lumbar normal and degenerative NP group are shown. The quantified results are shown on the right (*n* = 8). **d** The protein level of RCAN1.4 was higher than that of RCAN1.1 and progressively increased from the control group to the grade V group. **e** The upregulated RCAN1 and downregulated HIF-1α expression in a tail-looping disc degeneration rat model. Results of quantitative analysis of the percentage of positive cells among the NP cells are shown on the right (*n* = 4). All experiments were performed at least three times. Scale bar: 100 μm. *P < 0.05, **P < 0.01, ***P < 0.005.

We divided patients into the Pfirrmann grade I/II, III, IV, and V groups based on the Pfirrmann grading system^30^. Representative H&E, safranin O/fast green, and Alcian blue staining images of degenerative discs used to investigate the extent of degeneration are shown in Fig. 1a. We then detected gene expression of RCAN1.1, RCAN1.4, SOX9, type II collagen (Col2a1), aggrecan (ACAN), MMP13, and MMP3 in the four different groups using RT-qPCR (Fig. 1b). There was no obvious difference in the gene expression of RCAN1.1 or RCAN1.4 between the IDD group (grade IV and grade V) and the mild IDD group (grades I/II and grade III). Nevertheless, the RCAN1 protein level was visibly increased in the IDD group by immunohistochemistry (Fig. 1c). Furthermore, western blot assay showed that the protein level of RCAN1.4 was higher than that of RCAN1.1 and progressively elevated from the control group to the grade V group (Fig. 1d). In addition, we demonstrated upregulated RCAN1 and downregulated HIF-1α levels in a tail-looping disc degeneration model in rats (Fig. 1e). These data indicated that RCAN1.4 is strongly associated with disc degeneration.

### RCAN1.4 was downregulated under hypoxic conditions

**Fig. 2:**
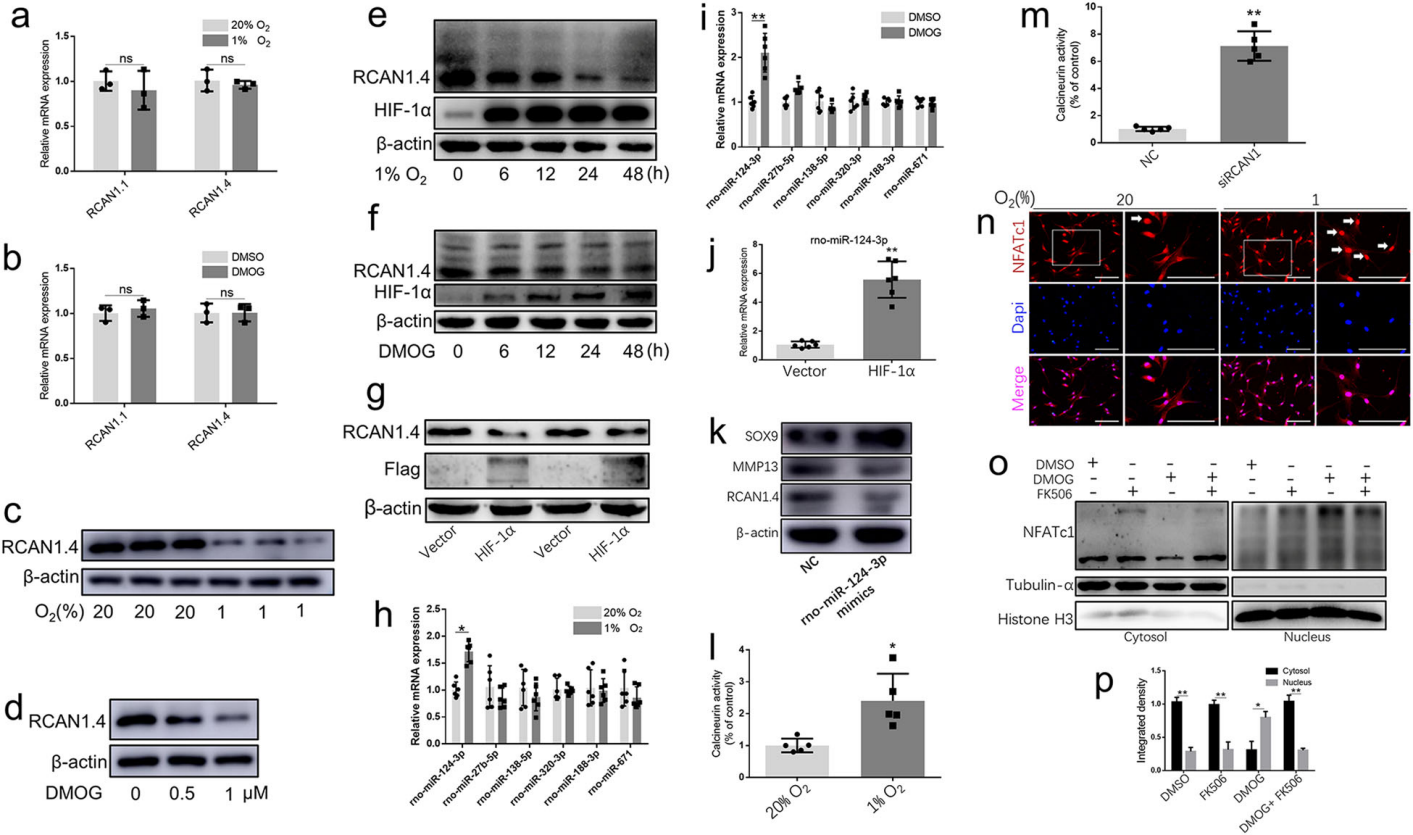
Hypoxia activated the calcineurin/NFAT signaling pathway through suppression of RCAN1.4. **a** Relative gene expression of RCAN1.1 and RCAN1.4 in human NP cells under hypoxia or **b** DMOG treatment compared with that in the normoxia group. **c** Relative protein level of RCAN1.4 in human NP cells under hypoxia or **d** DMOG treatment compared with that in the normoxia group. **e** The RCAN1.4 protein level was visibly decreased in a time-dependent manner under hypoxia or **f** DMOG treatment. **g** The RCAN1.4 protein level in human NP cells was reduced with HIF-1α overexpression. **h** Several microRNAs targeting the RCAN1 were selected. Under hypoxia or **i** DMOG treatment, rno-miR-124-3p was slightly increased in rat NP cells. **j** Overexpression of HIF-1α in rat NP cells upregulated rno-miR-124-3p. **k** An rno-miR-124-3p mimic could suppress RCAN1.4 expression in rat NP cells. **l** The activity of calcineurin in rat NP cells was upregulated under hypoxia or **m** RCAN1.4 siRNA treatment. **n** Immunofluorescence and **o** western blotting demonstrated the nuclear translocation of NFATc1 was obviously evaluated in rat NP cells under hypoxia. **p** The quantified results of the assessment of nuclear translocation of NFATc1 are shown. All experiments were performed at least three times. Scale bar: 100 μm. *P < 0.05, **P < 0.01, ***P < 0.005.

Aggravation of IDD has been associated with downregulated expression of HIF-1α but not HIF-2α^11,33^. Thus, we detected RCAN1.4 expression under conditions of hypoxia or HIF-1α overexpression to investigate the relationship between RCAN1.4 and relative hypoxia in NP cells. Gene expression of RCAN1.1 or RCAN1.4 was not significantly altered in human NP cells under hypoxic conditions or DMOG (a PHD inhibitor) treatment for 48 h (Fig. 2a and b). However, the RCAN1.4 protein level in human NP cells was visibly decreased in a dose- and time-dependent manner under hypoxia or DMOG treatment (Fig. 2c–f). In addition, the RCAN1.4 protein level in human NP cells was reduced with the overexpression of HIF-1α (Fig. 2g). To further investigate the mechanism of RCAN1.4 downregulation, we stimulated human NP cells with MG132 (a proteasome inhibitor) or leupeptin (a lysosome inhibitor). As shown in Supplemental Fig. 1a and b, the change in RCAN1.4 expression was not reversed when the proteasome or lysosomal pathway was blocked. Furthermore, the downregulation of RCAN1 under hypoxia was not dependent on the ubiquitination degradation pathway (Supplemental Fig. 1c). We also predicted the microRNA-binding sites targeting RCAN1 and selected several microRNAs. Under hypoxia or DMOG treatment, rno-miR-124-3p was slightly increased (Fig. 2h and i). In addition, overexpression of HIF-1α upregulated the rno-miR-124-3p level (Fig. 2j), and rno-miR-124-3p mimic suppressed RCAN1.4 expression (Fig. 2k). Together, these results show that RCAN1.4 was downregulated by increasing levels of rno-miR-124-3p under hypoxic conditions.

### Downregulated RCAN1 was accompanied with the activation of calcineurin/NFAT under hypoxic conditions

As expected, the activity of calcineurin was upregulated under hypoxia (Fig. 2l). Because RCAN1.4 can specifically bind calcineurin at or near the calcineurin A catalytic domain and downregulate calcineurin activity^14,15^, we knocked down RCAN1.4 by using RCAN1.4 siRNA and detected the activity of calcineurin in rat NP cells. Notably, calcineurin A was activated in rat NP cells transfected with RCAN1.4 siRNA (Fig. 2m). Furthermore, IF and western blotting demonstrated that the nuclear translocation of NFATc1 was obviously increased in rat NP cells under hypoxia (Fig. 2n–p), indicating that the calcineurin/NFAT signaling pathway is activated in hypoxic NP cells, mainly by the downregulation of RCAN1.4.

### RCAN1.4-calcineurin/NFAT signaling pathway altered ECM synthesis and remodeling

**Fig. 3:**
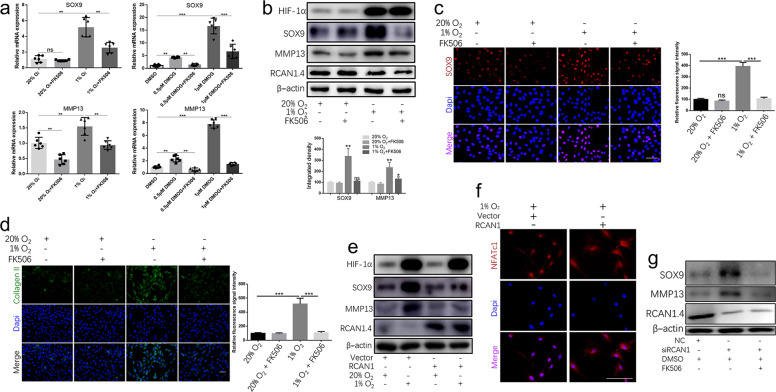
RCAN1.4 the ECM synthesis and remodeling by regulating the calcineurin/NFAT signaling pathway. **a** Relative SOX9 and MMP13 gene expression in rat NP cells treated with or without FK506 (a calcineurin inhibitor) and stimulated with hypoxia or DMOG treatment. **b** Western blot analysis of rat NP cells treated with or without FK506 and stimulated with hypoxia or DMOG treatment. The quantified results are shown below. **c** Immunofluorescence analysis of SOX9 and **d** type II collagen in rat NP cells treated with or without FK506 and stimulated with hypoxia or DMOG treatment for 48 h. Results of the quantification of SOX9 and type II collagen expression are shown on the right. **e** Western blot analysis of rat NP cells treated with vector or RCAN1.4 plasmid and stimulated with hypoxia or DMOG treatment. **f** Immunofluorescence demonstrated that RCAN1.4 overexpression suppressed the nuclear translocation of NFATc1 in rat NP cells under hypoxia. **g** Relative expression of SOX9 and MMP13 in rat NP cells treated with or without FK506 and stimulated with negative control siRNA or 100 nM RCAN1.4 siRNA. All experiments were performed at least three times. Scale bar: 100 μm. *P < 0.05, **P < 0.01, ***P < 0.005.

To reveal the function of the RCAN1.4-calcineurin/NFAT signaling pathway in IDD, we used RCAN1.4 siRNA, RCAN1.4 overexpression plasmid, or FK506 (a calcineurin inhibitor) in rat NP cells under hypoxia. As shown in Fig. 3a and b, hypoxia-induced upregulation of SOX9 and MMP13 was restrained by FK506. Furthermore, an immunofluorescence experiment showed that nuclear translocation of SOX9 and expression of type II collagen under hypoxia were sharply reduced by FK506 (Fig. 3c and d). Furthermore, RCAN1.4 overexpression reversed hypoxia-induced expression of SOX9 and MMP13 (Fig. 3e) and inhibited the hypoxia-induced nuclear translocation of NFATc1 (Fig. 3f). In addition, RCAN1.4 knockdown by RCAN1.4 siRNA promoted the expression of SOX9 and MMP13, but their high expression levels were strongly suppressed by FK506 (Fig. 3g). Together, these data show that calcineurin/NFAT activation by hypoxia-induced downregulation of RCAN1.4 promotes ECM synthesis and remodeling.

### **Nuclear translocation of NFATc1 promoted SOX9 and MMP13 promoter activity**

**Fig. 4:**
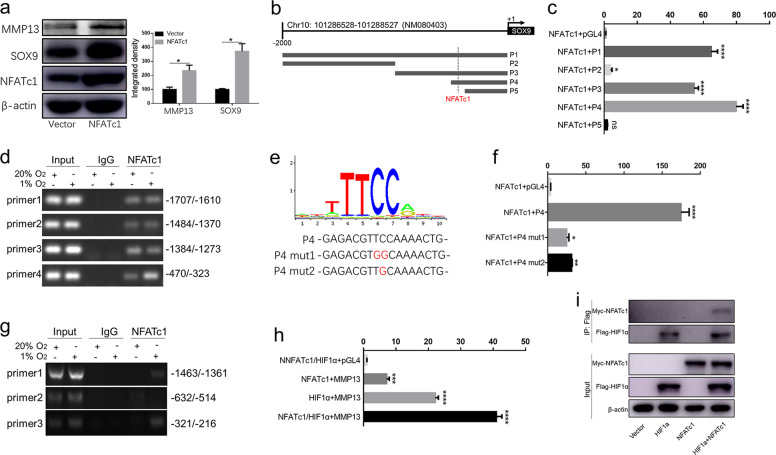
NFATc1 directly bound the SOX9/MMP13 promoter region. **a** Overexpression of NFATc1 upregulated SOX9 and MMP13 expression in rat NP cells. **b** Five constructs derived from 2000 bp upstream of the rat SOX9 promoter. **c** NFATc1-drived luciferase activity of the P1, P2, P3, P4, and P5 fragments in rat NP cells. **d** NFATc1 increasingly bound the SOX9 promotor site (bp −470 to −323) by ChIP assay. **e** Mutations in the putative NFATc1-binding site of the P4 fragment. **f** The P4 mutant decreased NFATc1-drived luciferase activity in rat NP cells. **g** NFATc1 could also bind sites in the MMP13 promotor (bp −1463 to −1361 and −321–216). **h** Similarly, NFATc1 promoted the transcriptional level by binding the MMP13 promotor region. **i** Hypoxia caused accumulation of the HIF-1α and NFATc1 protein complex. All experiments were performed at least three times. *P < 0.05, **P < 0.01, ***P < 0.005.

To illuminate the mechanism by which hypoxia promotes the transcription of SOX9 and MMP13, we first demonstrated that overexpression of NFATc1 upregulated SOX9 and MMP13 expression (Fig. 4a). We then cloned their promoter into a luciferase reporter plasmid. We first predicted putative NFATc1-binding sites within the SOX9 and MMP13 promoter regions with a length of 2000 bp with JASPAR: http://jaspar.genereg.net. We then generated five constructs derived from the 2000 bp upstream of the rat SOX9 promoter (Fig. 4b). We detected robust NFATc1-drived luciferase activity with the P1, P3, and P4 fragments, but not the P2 and P5 fragments, in NP cells (Fig. 4c), indicating the presence of a positive regulatory element between P4 and P5. Further, to verify that NFATc1 regulates endogenous SOX9 transcription, we conducted a ChIP assay in NP cells. Surprisingly, NFATc1 increasingly bound the SOX9 promotor site (bp −470 to −323) in NP cells under hypoxia, whereas other binding sites (bp −1717 to −1610, −1484 to −1370 and −1384 to −1273) were not elevated (Fig. 4d). Furthermore, we introduced mutations in the putative NFATc1-binding site of the P4 fragment (P4 mut1 and mut2) (Fig. 4e) and showed that P4 mutation decreased luciferase activity (Fig. 4f). Similarly, a ChIP assay demonstrated that NFATc1 could also bind sites in the MMP13 promotor (bp −1463 to −1361 and −321 to −216) in NP cells under hypoxia, while binding at the other binding site (bp −632 to −514) was not elevated (Fig. 4g). Surprisingly, we revealed that the interaction of HIF-1α and NFATc1 positively regulated MMP13 transcription (Fig. 4h). In addition, accumulation of the of HIF-1α and NFATc1 protein complex increased under hypoxia, which might facilitate their gene transcription (Fig. 4i).

### The role of the RCAN1.4-calcineurin/NFAT signaling pathway in an ex vivo model of rat discs

**Fig. 5:**
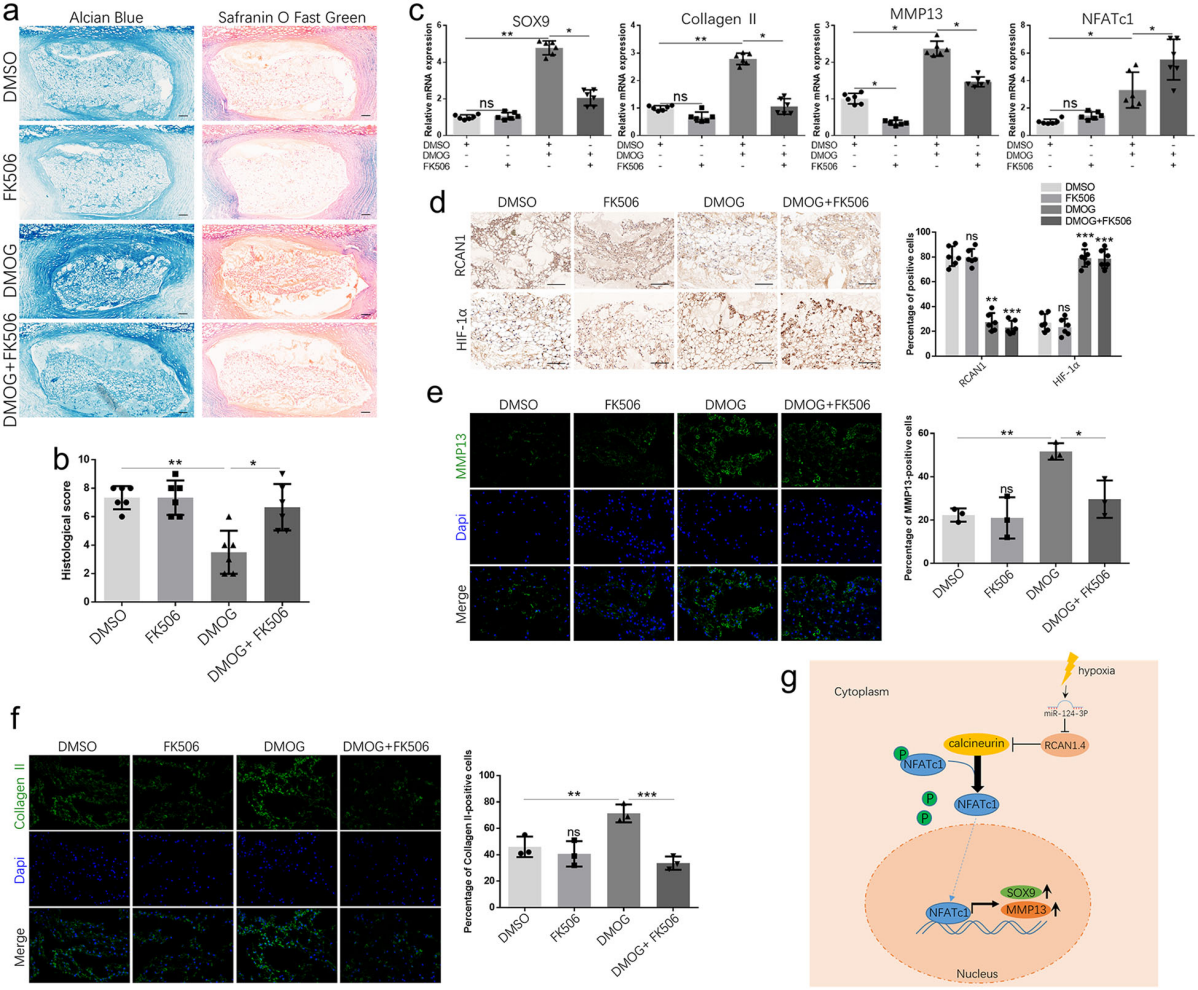
The role of RCAN1.4-calcineurin/NFAT in an ex vivo rat disc model. **a** Safranin O/fast green and Alcian blue staining of sections of discs treated with or without DMOG (1 μm), or FK506 (1 μm) for 14 days was carried out. **b** Histological scores for discs in different groups. **c** Relative SOX9, type II collagen, MMP13, and NFATc1 gene expression in NP tissues from different groups. **d** IHC analysis of RCAN1 and HIF-1α expression in NP disc sections. Results of quantitative analysis of the percentage of positive cells among NP cells are shown on the right. **e** Immunofluorescence analysis of MMP13 and **f** type II collagen in NP disc sections. Results of quantitative analysis of the percentage of positive cells among NP cells are shown on the right. **g** Schematic representation of major molecular pathway was presented. *n* = 6. Scale bar: 100 μm. *P < 0.05, **P < 0.01, ***P < 0.005.

To investigate the potential protective effects of RCAN1.4-calcineurin/NFAT in IVDDs, we generated an ex vivo model of rat lumbar discs. Safranin O/fast green and Alcian blue staining demonstrated the disc ECM in the control group was slightly degraded (Fig. 5a), as previously described^34^. In addition, DMOG treatment attenuated disc degeneration (Fig. 5a). FK506 (a calcineurin inhibitor) counteracted the effect of DMOG on discs (Fig. 5a and b). After DMOG treatment for 14 days, SOX9, type II collagen, MMP13, and NFATc1 expression was significantly increased (Fig. 5c). However, when discs were costimulated with FK506 and DMOG, SOX9, type II collagen, MMP13 expression was partially reversed to the levels in control cells (Fig. 5c). Immunohistochemistry showed decreased RCAN1 and increased HIF-1α expression after DMOG or FK506 treatment (Fig. 5d). In addition, immunofluorescence revealed that DMOG-induced MMP13 and type II collagen expression could be suppressed by FK506 treatment (Fig. 5e and f). Finally, schematic representation of major molecular pathway was presented (Fig. 5g).

## Discussion

Disc degeneration classification is carried out through subscoring of the following major subtissues: the NP, AF, and CEP^35,36^. If any of these structures is damaged or degenerated, the hypoxic environment of the disc will be destroyed. Understanding the underlying mechanisms that regulates ECM synthesis and the remodeling of disc cells under hypoxia is vital for future treatment. In this study, we first demonstrated that RCAN1.4 is positively associated with disc degeneration and that hypoxia-induced downregulation of RCAN1.4 activated the calcineurin/NFAT signaling pathway to facilitate SOX9 and MMP13 expression. Furthermore, NFATc1 could directly bind the promotor regions of SOX9 and MMP13. In addition, a whole disc ex vivo model showed that the RCAN1.4-calcineurin/NFAT signaling pathway is critical for the hypoxic IVD phenotype under hypoxia.

RCAN1.4 has a wide range of biological roles, including protecting against calcium-mediated oxidative stress^37^, cardiac hypertrophy^38^, and vascular endothelial growth factor-mediated signaling during angiogenesis^39^. In addition, RCAN1 is more highly expressed than RCAN2 or RCAN3 in NP cells (data not shown). Therefore, we assumed that RCAN1 has a vital role in the IVD phenotype. Nevertheless, evidence regarding the role of RCAN1 in degenerative disc diseases is somewhat limited. We first showed that RCAN1.4 was upregulated with an increasing degree of disc degeneration. Although smaller vessels can infiltrate the cartilage endplates and outer AF layers, they cannot infiltrate the NP^5^. As a result, NP tissues under physiological conditions are a hypoxic microenvironment^7^. Cancer cells are also under a hypoxic microenvironment, and low expression levels of RCAN1.4 can promote the proliferation, migration, and invasive activity of cancer cells by promoting nuclear translocation of NFAT^17^. These studies indicated that RCAN1.4 participates in the hypoxic adaption of the IVD. Although calcineurin is activated by increased intracellular calcium^40^, we found that hypoxia inhibited RCAN1.4 protein expression, thus activating the calcineurin/NFAT signaling pathway and maintaining the hypoxic IVD phenotype. Degradation of RCAN1 was shown to be mediated by the ubiquitin-proteasome pathway^41^. However, our study demonstrated that downregulation of RCAN1.4 under hypoxia was not dependent on the ubiquitination degradation pathway. Nevertheless, we have not clarified the mechanism of RCAN1.4 downregulation, which might involve microRNA regulation.

Mature NP cells are small, chondrocyte-like cells sparsely distributed throughout the disc^42^. The transcription factor SOX9 is involved in chondrogenesis^43^. A recent study showed that a CoCl_2_-simulated hypoxic environment significantly increased the expression levels of calcineurin in mouse skeletal muscles^44^. Although hypoxia resulted in HIF-1α accumulation, enhancing SOX9-mediated expression of the target gene type II collagen in femoral chondrocytes^26^, it was still unclear whether the calcineurin/NFAT signaling pathway is involved in the regulation of SOX9. Interestingly, FK506, a calcineurin inhibitor, suppressed the nuclear translocation of NFATc1, thus restraining SOX9 transcription and expression in NP cells. We detected the expression level of NFAT family members and found a high level of NFATc1 expression (data not shown). In addition, NFATc1 was positively associated with upregulated SOX9 in the pancreas and tracheal cartilage^23,24^. We further revealed that NFATc1 enhanced the promoter activity of SOX9. By mutating the vital NFATc1-binding site in the SOX9 promotor region, we then determined that its primary binding site is from bp −367 to −357 on the 2000 bp upstream of the rat SOX9 promoter. It is possible that hypoxia resulted in accumulation of the HIF-1α and NFATc1 protein complex in hypoxic NP cells, assisting gene transcription.

PHD2 deficiency can activate the calcineurin/NFATc1-dependent pathway^21^, indicating that HIF-1α is possibly involved in activating the calcineurin/NFATc1 pathway. We found elevated calcineurin activity under hypoxia or RCAN1.4 siRNA transfection, further promoting the nuclear translocation of NFATc1. Although independent silencing of calcineurin A (PPP3CA) expression stimulated HIF transcriptional activity under hypoxia^45^, we could not determine the regulated relationship between calcineurin and HIF-1α based on our results. Hypoxia-induced mitogenic factor overexpression increased the cytosolic Ca^2+^ concentration and activated the calcineurin/NFAT pathways in cardiac hypertrophy^29^. Here, we detected the cytosolic Ca^2+^ concentration in hypoxic NP cells and found no noticeable change in the cytosolic Ca^2+^ concentration of NP cells (data not shown). Nevertheless, we showed the activation and inactivation of the calcineurin/NFATc1 signaling pathway by overexpression or blockade of RCAN1.4, respectively. Calcium influx could be associated with the RCAN1.4 protein level, but the relationship between calcium and RCAN1.4 in NP cells still requires further confirmation.

Hypoxia strongly promotes ECM synthesis, after which the ECM is remodeled and better organized to maintain homeostasis in the disc. We found slightly elevated levels (~1.6-fold) of MMP13, a marker of ECM remodeling, under 1% O_2_. A study reported that the interaction between β-catenin and HIF-1α inhibited MMP13 expression and prohibited articular cartilage damage in mice^46^. Hypoxia suppressed serum deprivation-induced ECM degradation of NP cells through the JNK and NF-κB pathways^47^. Hypoxic isolation-expansion of human NP cells was important to achieve better regenerative cells for later cultivation or cell transplantation^48^. However, hypoxia was also shown to alter the gut microbiota, resulting in the senescence of bone marrow mesenchymal stem cells^49^. Therefore, hypoxia might be a “double-edged sword”. We believe that hypoxia slightly remodels the ECM while hypoxia strongly promotes ECM synthesis in NP cells. Based on the results of the luciferase assay, NFATc1 strongly bound the MMP13 promotor region, thus upregulating MMP13 transcription. MMP13 expression could also be suppressed by FK506.

In conclusion, RCAN1.4 is involved in the hypoxia-induced IVD phenotype mainly through its regulation of the calcineurin/NFATc1 signaling pathway in NP cells. The calcineurin/NFATc1 signaling pathway is crucial for ECM synthesis and remodeling in discs. Our study shows that RCAN1.4 could be a potential therapeutic target for degenerative disc diseases. Further studies might focus on the effect of RCAN1.4 on AF or the cartilage endplates.

## Supplementary information

Supplemental material
