# Supplementary material for: The RCAN1.4-calcineurin/NFAT signaling pathway is essential for hypoxic adaption of intervertebral discs
Source: Exp Mol Med. 2020 May 29;52(5):865–75. doi: 10.1038/s12276-020-0441-x (PMC7272636; doi:10.1038/s12276-020-0441-x)
Supplement: Supplementary file 1 — Supplemental material [file 12276_2020_441_MOESM1_ESM.pdf]

Supplemental Figure legends

Supplemental Figure 1

(a)RCAN1.4 was not reversed in human NP cells by blocking the proteasome (MG132, a proteasome inhibitor) or (b) lysosomal (leupeptin, a lysosome inhibitor) pathway. (c) Downregulation of RCAN1.4 in human NP cells was not dependent on ubiquitination degradation pathway under hypoxia.

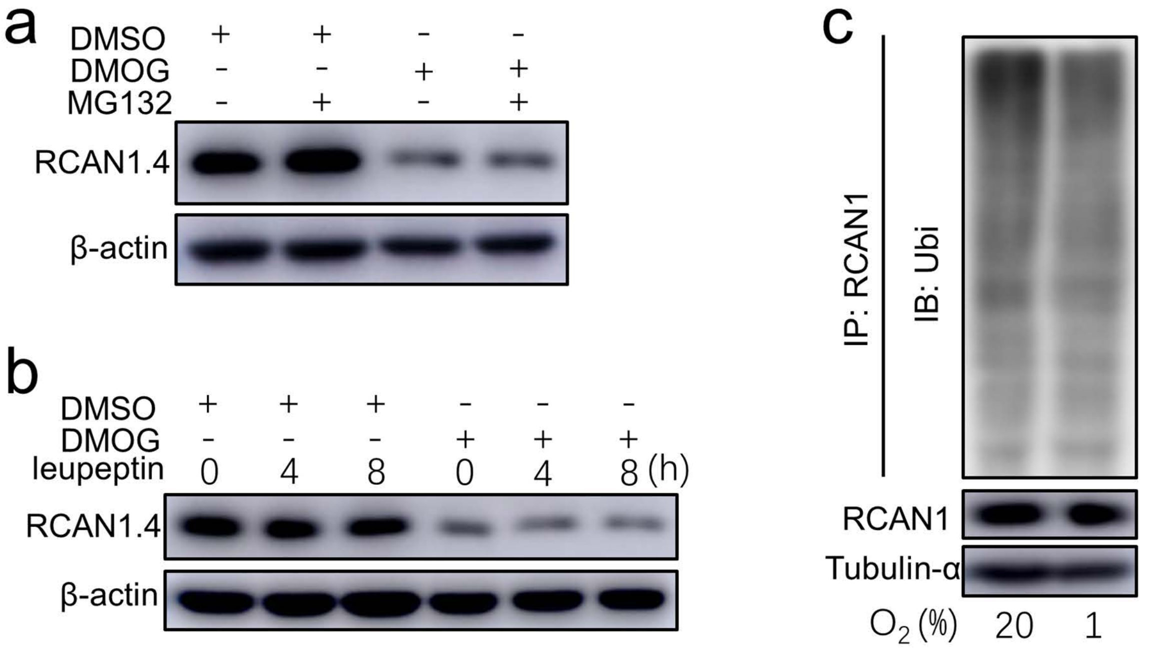
a
DMSO + + - -
DMOG - - + +
MG132 - + - +
RCAN1.4
β-actin
b
DMSO + + + - - -
DMOG - - - + + +
leupeptin 0 4 8 0 4 8 (h)
RCAN1.4
β-actin
c
IP: RCAN1
IB: Ubi
RCAN1
Tubulin-α
O2 (%) 20 1

Supplemental Table 1. Patients' Clinical Characteristics

Characteristic	Control Group	Study Group
Number	28	24
Age (yr)	39.36(22-57)	56.74 (35-69)
Sex(male/female)	13/15	12/12
Grade Ⅰ/ Ⅱ	11	
Grade Ⅲ	17	
Grade Ⅳ		14
Grade Ⅴ		10

Supplemental Table 2 Primer sequences

Gene	Stream	Sequence
Rat Sox9	Forward	5'-GGAATGTTTCAGCAGCCAAT-3'
	Reverse	5'-TGGTGTTCTGAGAGGCACAG-3'
Rat Col2a1	Forward	5'-CTGGAAAAGCTGGTGAAAGG-3'
	Reverse	5'-GGCCTGGATAACCTCTGTGA-3'
Rat RCAN1	Forward	5'-TGTGGCAAACGGTGATGTCT-3'
	Reverse	5'-CAGACAGGGGGTTGCTGAAG-3'
Rat NFATc1	Reverse	5'-AATAACCAGCCCCGTCCAAG-3'
	Reverse	5'-GGTCAGAGCTGGCTCAAAGT-3'
Rat MMP13	Forward	5'-ACTGAGAGGCTCCGAGAAATG-3'
	Reverse	5'-GAACCCCGCATCTTGGCTT-3'
Rat β-actin	Forward	5'-CTATGAGGGTTACGCGCTCC-3'
	Reverse	5'-ATGTCACGCACGATTTCCCT-3'
Human SOX9	Forward	5'-AAGGACCACCCGGATTACAAG-3'
	Reverse	5'-GTTGGGGGAGATGTGCGTC-3'
Human Col2a1	Forward	5'-GCTCCTGCCGTTTCGCTG-3'
	Reverse	5'-ATTATACCTCTGCCCATCCTGC-3'
Human RCAN1.1	Forward	5'-TGGAGCTTCATTGACTGCGA-3'
	Reverse	5'-ACGTCCTAAAGAGGGACTCA-3'
Human RCAN1.4	Forward	5'- TTTAGCTCCCTGATTGCCTGT-3'
	Reverse	5'-AAAGGTGATGTCCTTGTCATACG-3'
Human MMP13	Forward	5'-TCGGCCACTCCTTAGGTCTT-3'
	Reverse	5'-AAGTGGCTTTTGCCGGTGTA-3'
Human MMP3	Forward	5'-CACTCACAGACCTGACTCGG-3'
	Reverse	5'-GAGTCAGGGGGAGGTCCATA-3'
Human Aggrecan	Forward	5'-CTTCCGCTGGTCAGATGGAC-3'
	Reverse	5'-CGTTTGTAGGTGGTGGCTGT-3'
Human β-actin	Forward	5'-TGGAACGGTGAAGGTGACAG-3'
	Reverse	5'-AACAACGCATCTCATATTTGGAA-3'

Supplemental Table 3 Histological score of degenerative discs

Histological score of degenerative discs
I. Cellularity of the anulusfibrosus
Grade:
1. Fibroblasts comprise more than 75% of the cells
2. Neither fibroblasts nor chondrocytes comprise more than 75% of the cells
3. Chondrocytes comprise more than 75% of the cells
II. Morphology of the anulusfibrosus
Grade:
1. Well-organized collagen lamellae without ruptured or serpentinefibers
2. Inward bulging, ruptured or serpentine fibers in less than one third of the annulus
3.Inward bulging, ruptured or serpentine fibers in more than one third of the annulus
III. Border between the anulusfibrosus and nucleus pulposus
Grade:
1. Normal, without any interruption
2. Minimal interruption
3. Moderate or severe interruption
IV. Cellularity of the nucleus pulposus
Grade:
1. Normal cellularity with stellar shaped nuclear cells evenly distributed throughout the nucleus
2. Slight decrease in the number of cells with some clustering
3. Moderate or severe decrease (>50%) in the number of cells with all the remaining cells clustered and separated by dense areas of proteoglycans
V. Morphology of the nucleus pulposus
Grade:
1. Round, comprising at least half of the disc area in mid sagittal sections
2. Rounded or irregularly shaped, comprising one quarter to half of the disc area in mid sagittal sections
3. Irregularly shaped, comprising less than one quarter of the disc area in mid sagittal sections
